# The necessarity of treatment for small gastric subepithelial tumors (1–2 cm) originating from muscularis propria: an analysis of 972 tumors

**DOI:** 10.1186/s12876-022-02256-3

**Published:** 2022-04-11

**Authors:** Jinlong Hu, Xinzhu Sun, Nan Ge, Sheng Wang, Jintao Guo, Xiang Liu, Guoxin Wang, Siyu Sun

**Affiliations:** Department of Gastroenterology, Shengjing Hospital of China Medical University, Shenyang, 110004 China

**Keywords:** Gastric subepithelial tumor, Gastrointestinal stromal tumor, Endoscopic ultrasound

## Abstract

**Background and objectives:**

Up till now, there are still controversies about the specific indication of endoscopic resection for small gastric subepithelial tumors (gSETs) originating from muscularis propria. We aimed to investigate the safety of endoscopic resection and postoperative pathology analysis.

**Method:**

The patients with primary small gSETs originating from muscularis propria, treated by endoscopic resection in the endoscopic center of Shengjing Hospital between January, 2011 and September, 2019 were enrolled. The complete resection rate, adverse events and clinicopathological features were recorded.

**Result:**

A total of 936 patients with 972 gastric SETs ≤ 2 cm originating from muscularis propria were included in our study. All the lesions were successfully treated by endoscopic resection. Nearly half of lesions were proved to be gastrointestinal stromal tumor (GIST) [n = 411 (42.3%)] according to postoperative pathology. All the objects were further subdivided into 2 groups, ≤ 1 cm, > 1 and ≤ 2 cm gSETs. The risk of gastric GIST of intermediate/high risk in the group (> 1 and ≤ 2 cm gSETs) is 8.41 times as that of gastric GIST in the group (the size of gastric ≤ 1 cm gSETs) (*P* < 0.05).

**Conclusion:**

Endoscopic resection is a safe and effective treatment for small gSETs. gSETs (1–2 cm) is more risky than gSETs (≤ 1 cm) and should be resected. This should be evaluated by further studies.

## Introduction

As reported by earlier literature, the incidence of gastric subepithelial tumors (gSETs) for routine gastroscope examination was about 0.36–1.94% [1–3]. Gastrointestinal stromal tumors (GISTs), usually originating from the muscularis propria [4], are the most common type of gSETs [5] and considered potentially malignant. According to the latest guidelines published by European Sarcoma Network Working Group [6] and National Comprehensive Cancer Network [7, 8], surveillance and follow-up, other than positive excision, are recommended for gSETs ≤ 2 cm without high-risk endoscopic ultrasonography (EUS) features or related clinical symptoms, since they are of very low risk of malignancy and metastasis. However, a portion of scholars hold that small gSETs, especially GISTs of intermediate or high risk, should be resected as soon as detected in order to make a confirm diagnosis and to avoid further malignancy [9]. Moreover, endoscopic procedures are extremely suitable for these gSETs [10].

Several studies have proved the efficacy and safety of endoscopic resection for small gSETs [11–13]. However, few studies have concentrated on necessity for the resection of small gSETs. The current criterion for small gSETs resection may not find all the high risky lesions. In the present study, we used a larger sample size to analyze safety and necessarity of endoscopic management and clinicopathological data of small gSETs ≤ 2 cm originating from the muscularis propria.

## Patients and methods

### Patients

In the present study, we included the demographic and clinicopathological results of 936 consecutive patients with 972 primary small gastric SETs originating from muscularis propria, treated by endoscopic resection in the endoscopic center of Shengjing Hospital of China Medical University between January, 2011 and September, 2019. If the lesion involved more layers, it was excluded. All the patients in the present study asked to remove gastric lesion. All patients were evaluated by computed tomography and endoscopic ultrasound (EUS) (The maximum diameter < 2.0 cm). The EUS characteristics of the lesion, endoscopic treatment, adverse events and postoperative pathology were analyzed. Furthermore, we analyzed whether the size of GIST larger than 1 cm was responsible for the postoperative pathology to be intermediate or high-risk according to the modified NIH risk stratification via Chi-Squared Test. All included patients were provided with written informed consent to undergo endoscopic resection and this study was supported by the institutional review board of Shengjing hospital.

### Endoscopic treatment

All the procedures were performed under general anesthesia. The details of band ligation and resection, endoscopic submucosal dissection, endoscopic full-thickness resection and submucosal tunneling endoscopic resection were described in various studies [14–16]. Postoperative histology evaluation and immunohistochemistry was used to identify tumor type.

## Results

936 patients with 972 gastric SETs ≤ 2 cm originating from muscularis propria were included in our study (Table 1). Among the 936 patients, 213 were male (22.8%), 723 were female (77.2%). The mean age was 53.6 ± 10.2 years (range 16–78 years). The median value for maximal tumor size was 9.6 ± 3.9 mm (range 3–20 mm). All the lesions were successfully treated by endoscopic resection. Three patients experienced postoperative bleeding, which were successfully managed by endoscopic hemostasis. Two patients experienced postoperative perforation. One patient was treated by conservative treatment and one patient received surgery. Pathological diagnosis were leiomyoma [n = 485 (49.9%)], GIST [n = 411 (42.3%)], schwannoma [n = 20 (2.1%)], ectopic pancreas [n = 12 (1.2%)] and other tumors or tissues [n = 44 (4.5%)]. The details of patients with GIST were listed in Table 2. According to the modified NIH risk classification system, there were 376 cases (91.5%) of very low risk, 25 cases (6.1%) of low risk, 8 cases (1.9%) of intermediate risk and 2 cases (0.5%) of high risk. Only two patients with intermediate or high risk GIST had epigastric discomfort. Ultimately, we analyzed whether size > 1 cm was correlated with the postoperative pathology to be intermediate or high-risk according to the modified NIH risk stratification via Chi-Squared Test. Therefore, tumors are more likely to be of relatively higher risk when the sizes are larger than 1 cm. The OR value was 8.41, that is, the risk of gastric GIST of intermediate/high risk in the group (the size of gastric subepithelial tumor ranging 1–2 cm) is 8.41 times as that of gastric GIST in the group (the size of gastric subepithelial tumor less than 1 cm) (*P* < 0.05) (Table 3).

**Table 1:** Clinical characteristics of the 936 patients with 972 gastric SETs ≤ 2 cm

Age [year; mean ± SD; (range)]	53.6 ± 10.2 (16–78)
Gender [n (%)]	
Male	213 (22.8%)
Female	723 (77.2%)
Tumor diameter [mm; mean ± SD]	9.6 ± 3.9
Location [n (%)]	
Fundus	558 (57.4)
Corpus	390 (40.1)
Angle	3 (0.3)
Antrum	21 (2.2)
Pathological diagnosis [n (%)]	
Leiomyoma	485 (49.9)
GIST	411 (42.3)
Schwannoma	20 (2.1)
Ectopic pancreas	12 (1.2)
Vascular malformation/proliferation	8 (0.8)
Inflammatory tissue	17 (1.7)
Hamartoma	5 (0.5)
Glomus tumor	4 (0.4)
Neuroendocrine neoplasm	3 (0.3)
Calcified tissue	3 (0.3)
Collagenoma	2 (0.2)
Hemangioma	1 (0.1)
Fibroma	1 (0.1)
Endoscopic procedures [n (%)]	
BLR	418 (43)
ESD	344 (35.4)
EFTR	201 (20.7)
STER	9 (0.9)
Complete resection	972
Postoperative complications	
Perforation	2
Bleeding	3

*BLR* band ligation and resection, *ESD* endoscopic submucosal dissection, *EFTR* endoscopic full-thickness resection, *STER* submucosal tunneling endoscopic resection

**Table 2:** Clinical features and endoscopic procedure of 411 GISTs of 403 patients

Age [year; mean ± SD; (range)]	56.5 ± 8.6 (29–78)
Gender [n (%)]	
Male	115 (28.5%)
Female	288 (71.5%)
Tumor diameter [mm; mean ± SD]	9.7 ± 4.1
Location [n (%)]	
Fundus	288 (69.8)
Corpus	114 (18.7)
Antrum	9 (2.2)
Pathological diagnosis [n (%)]	
Very-low risk	376 (91.5)
Low risk	25 (6.1)
Intermediate risk	8 (1.9)
High risk	2 (0.5)
Endoscopic procedures [n (%)]	
BLR	159 (38.6)
ESD	118 (28.7)
EFTR	133 (32.4)
STER	1 (0.1)
EUS features	
Hypoechoic lesion	411
Irregular border	0
Cystic spaces	0
Ulceration	0
Heterogeneity	0

**Table 3:** Pathological characteristics according to tumor size

	> 1 and ≤ 2 cm	≤ 1 cm	Total
GISTs of intermediate/high risk (n_1_)	8	2	10
Gastric tumors other than GISTs of intermediate/high risk (n_2_)	310	652	962
Total	318	654	972
OR	8.41		
χ^2^	8.20 > χ^2^_0.05,1_ = 3.84, *P* < 0.05		

## Discussion

With the development of endoscopic techniques and improvement of health awareness, the detection rate of gSETs has increased in recent years [17]. On the basis of the biological behavior and cytological characteristics, gSETs are classified into non-neoplastic and neoplastic lesions [5]. Non-neoplastic SETs usually manifest as benign lesions, such as inflammation, cysts, ectopic pancreas, etc. On the contrary, a portion of neoplastic gSETs are malignant or potentially malignant lesions, such as GISTs. Among all the neoplastic SETs of digestive tract, GISTs are considered as the most common tumor and potentially malignant neoplasms originating from the interstitial cells of Cajal [18]. GISTs account for 1/5 of sarcoma of gastrointestinal tract and represent 1–2% of all gastrointestinal malignancies [19] and more than a half of them are located in the stomach [20, 21].

Based on EUS guideline [22], EUS is the most effective method for differentiating between an intramural and extramural lesion and for evaluating the characteristics, such as layer of origin, size, margin and echogenicity, of subepithelial lesions. But it is difficult to make differential diagnosis of gastric tumor originating from muscularis propria by EUS, especially for small lesion. Based on the big lesions, some authors concluded that high-risk EUS features provided evidence for diagnosis of malignant or potentially malignant GISTs, including irregular border, cystic spaces, ulceration, echogenic foci, and heterogeneity [23, 24]. Moreover, contrast-enhanced EUS can be used for differentiation [25] and intra-tumoral vessels observed in GISTs using contrast-enhanced EUS was proved to be correlated with higher malignant potential by the study of Yasunobu [26]. However, studies also showed that ultrasonographic features of smaller lesions might not have been as sensitive as those of larger ones [27]. In our study, EUS could not predict the potential malignancy of GISTs. Besides, a number of EUS-related sampling strategies, such as EUS-guided fine needle aspiration (EUS-FNA) [28–30], EUS-guided core needle biopsy [31], as well as the latest “ligate unroof biopsy” technique [32], have emerged to assist differential diagnosis between malignant GISTs and other congenital lesions or benign neoplasms, such as leiomyomas. Recent studies have reported high diagnostic yields of EUS-FNA applied in gSETs, particularly those originating from muscularis propria [33, 34]. However, confirmed diagnosis of gSETs (≤ 2 cm) may not rely on EUS-FNA alone due to difficulty to perform and low diagnostic yields when tumors are small [35]. Moreover, there would not be sufficient tissues obtained by biopsy for the assessment of mitotic or genetic mutation [36]. Due to heterogeneity of most tumors, pathologists cannot determine the level of mitoses of GISTs through samples gained by FNA or other biopsy strategies since the cellularity and the mitotic count of GISTs are inconsistent in different sites within the tumors.

Up till now, there are still controversies about the specific indication of endoscopic resection for small gastric gSETs originating from muscularis propria. According to the guidelines published by ESMO in 2014, the standard approach to the small, asymptomatic, upper gastrointestinal SETs < 2 cm is EUS assessment and then annual follow-up, reserving excision for tumors which increase in size or for patients who turn symptomatic [6]. The NCCN guidelines (2010) also suggested that, for patients without high-risk EUS features (i.e. irregular border, cystic spaces, ulceration, echogenic foci, and heterogeneity), a follow-up with EUS was recommended every 6–12 months [7, 8]. In other words, surveillance and follow-up, other than positive excision, are recommended for SETs < 2 cm by the guidelines. However, differentiation between potentially malignant GISTs and other benign or non-neoplastic lesions is extremely difficult by image method, especially for small lesions. In addition, some studies found small GISTs (< 2 cm) may be intermediate or high risk. Yang et al. reported that there were 7 cases (2.5%) of intermediate risk and 10 cases (3.6%) of high risk in gastric GISTs (< 2 cm) in their study [17]. Pang et al. reported that 9 patients (3.9%) were in intermediate risk group and 2 patients (0.9%) were in high risk group, in which the tumor size in both cases was less than 2 cm [11]. Gao et al. found EUS-suspected GISTs larger than 9.5 mm may be associated with significant progression [27]. In a study from Italy, in which 170 GISTs measuring 2 cm or smaller were analyzed, mitotic activity dramatically increased once the tumor size exceeded 1 cm, compared with tumors smaller than 1 cm [37]. In our study, the lesions (between 1 and 2 cm) was more risky than lesions (< 1 cm). The OR value was 8.41. So the lesion (> 1 cm) had more potential to become malignant lesion and should be resected. Except GISTs, some other tumors originating from muscularis propria, such as gastric glomus tumor, are also potentially malignant. So in our opinion, we should resect gastric submucosal tumor originating from muscularis propria, if the size is larger than 1 cm. In addition, the study found that 27.6% (16/58) patients experienced a severe psychological illness and seriously affected their quality of life [9]. Due to the possibility of potential malignancy or malignancy, many patients tolerated the psychological stress and preferred to receive endoscopic treatment, instead of a regular follow-up.

Endoscopic resection methods, such as endoscopic submucosal dissection, endoscopic submucosal excavation, endoscopic full-thickness resection [38] and band ligation and resection, are widely used in clinical practice to remove gSETs originating from muscularis propria, showing low incidence of complications and the same therapeutic effect as open surgery and laparoscopic surgery [11, 13, 39–41] In our study, all the lesions were successfully removed by endoscopic method and postoperative bleeding and perforation were managed by endoscopic methods or conservative treatment, expect for one postoperative perforation, which was managed by surgery in the early time. With the development of endoscopic equipments, postoperative perforation could be managed by endoscopic method and following conservative treatment [42].

In conclusion, endoscopic resection may be suitable for the management of patients with small gastric SETs (1–2 cm) in order to make confirm diagnosis, to accomplish curative treatment, and to avoid potential malignancy. As for gSETs (≤ 1 cm), endoscopic and/or radiological surveillance should be recommended. Further studies should be conducted to confirm it.

## Data Availability

The dataset supporting the conclusions of this article is available from the corresponding author on reasonable request.

## Abbreviations

gSETs: Gastric subepithelial tumors
GIST: Gastrointestinal stromal tumor
EUS-FNA: EUS-guided fine needle aspiration
EUS: Endoscopic ultrasonography
